# Caspase-3 in Brain Death Donors Is Associated with Reduced Primary Graft Dysfunction After Heart Transplantation

**DOI:** 10.3390/ijms26199434

**Published:** 2025-09-26

**Authors:** Lorena Herrador, José González-Costello, Jordi Niubo-Bosch, Laura Calatayud-Samper, Alba Maestro-Benedicto, Marta Farrero-Torres, Teresa Blasco-Peiro, Luis Almenar-Bonet, Zorba Blázquez-Bermejo, Iris Garrido-Bravo, Ferran Gran-Ipiña, Antonio Grande-Trillo, Nicolas Manito, Gabriel Moreno-Gonzalez

**Affiliations:** 1 Heart Failure and Heart Transplant Unit, Cardiology Department, Bio-Heart Cardiovascular Diseases Research Group, Hospital Universitari de Bellvitge, Universitat de Barcelona, L’Hospitalet de Llobregat, 08907 Barcelona, Spain; lherrador@bellvitgehospital.cat (L.H.);; 2 Bellvitge Biomedical Research Institute (IDIBELL), L’Hospitalet de Llobregat, 08908 Barcelona, Spain; 3 Centro de Investigación Biomédica en Red de Enfermedades Cardiovasculares (CIBERCV), 28029 Madrid, Spain; 4 Microbiology Department, Hospital Universitari de Bellvitge, L’Hospitalet de Llobregat, 08907 Barcelona, Spain; 5 Heart Failure and Heart Transplant Unit, Cardiology Department, Hospital de la Santa Creu i Sant Pau, 08025 Barcelona, Spain; 6 Heart Failure and Heart Transplant Unit, Cardiology Department, Hospital Clínic Barcelona, 08036 Barcelona, Spain; 7 Heart Failure and Heart Transplant Unit, Cardiology Department, Hospital Universitario Miguel Servet, 50009 Zaragoza, Spain; tblascop@gmail.com; 8 Heart Failure and Heart Transplant Unit, Cardiology Department, Hospital Universitario y Politécnico La Fe, 46026 Valencia, Spain; 9 Heart Failure and Heart Transplantation Unit, Cardiology Department, Hospital General Universitario Gregorio Marañón, 28007 Madrid, Spain; 10 Heart Failure and Heart Transplantation Unit, Cardiology Department, Hospital Universitario Virgen de la Arrixaca, 30120 Murcia, Spain; 11 Pediatric Heart Transplant Unit, Pediatric Cardiology and Pediatric Cardiac Surgery Department, Hospital Vall Hebron, 08035 Barcelona, Spain; 12 Heart Failure and Heart Transplantation Unit, Cardiology Department, Hospital Universitario Virgen del Rocío, 41013 Sevilla, Spain; 13 Intensive Care Unit, Hospital Universitari de Bellvitge, L’Hospitalet de Llobregat, 08907 Barcelona, Spain

**Keywords:** brain death donor, primary graft disfunction, allograft rejection, inflammatory mediators, Caspase-3, cell death process, mitochondrial DNA

## Abstract

Primary graft dysfunction (PGD) remains a major cause of early morbidity and mortality after a heart transplant (HTx). Understanding the donor-related mechanisms involved may help improve organ selection and post-HTx outcomes. This study aimed to explore the association between the donor serum biomarkers of cell death and inflammation and the incidence of PGD and rejection in HTx recipients. We conducted a retrospective, multicenter observational study of brain-dead (DBD) heart donors and corresponding recipients between 2013 and 2019. Donor blood samples were analyzed for inflammatory cytokines, cell death-related proteins, and mitochondrial (mtDNA) and genomic DNA (gDNA). A total of 39 donor–recipient pairs were included. Sixteen recipients developed severe PGD, and five experienced ≥2R cellular rejection. Donors whose recipients developed PGD had significantly lower serum Caspase-3 levels compared to those without PGD (391.6 [101.8–1003.3] vs. 65.3 [40.2–163.3] pg/mL; *p* = 0.04). A trend toward lower mtDNA/gDNA ratio was also observed in the same group (10.5 [5.4–24.6] vs. 6.5 [3.3–10.7]; *p* = 0.067). Lower Caspase-3 levels in donor serum were significantly associated with the development of severe PGD in recipients. This may suggest that the sublethal activation of apoptotic pathways in the donor could play a protective role, potentially conditioning the graft to tolerate ischemic injury.

## 1. Introduction

Heart transplantation (HTx) remains the gold standard treatment for advanced heart failure. Post-transplant 1-year survival is around 80–90%, according to the registries [1,2] but these early outcomes are significantly impacted by primary graft dysfunction (PGD) and rejection. PGD remains the primary cause of early mortality after HTx despite the widespread use of mechanical circulatory support (MCS) to treat it. Its incidence and mortality range from 16–36% and 19–28%, respectively [3,4,5]. Several factors in both the donor and recipient have been associated with the development of PGD4 and there is a growing interest in discovering donor biomarkers that may predict PGD [6,7].

An intense activation of the sympathetic nervous system known as “autonomic storm” occurs after brain death [8], causes marked vasoconstriction followed by hypotension and decrease in cardiac output [9]. These hemodynamic alterations translate into histological changes with the appearance of contraction bands and cardiomyocyte necrosis [8]. Cytokine gene expression increases after brain death in animal models and clinical studies [10,11,12,13,14].

Some studies have assessed the association between pre-operative inflammatory biomarkers in brain death (DBD) donors and recipient outcome, yielding controversial results [15]. High donor inflammatory biomarkers such as IL-6, IL-8, TNFα or IL-10 have been related to worse recipient heart function and survival [13,16]. However, despite evidence of potential deleterious effect of a pro-inflammatory environment, donor treatment with steroids has not demonstrated its utility in this context [17,18]. On the other hand, a correlation between donor higher concentrations of soluble necrosis factor receptors, IL-10 and IL-6, and reduced hospitalization times in recipients has also been described [19]. However, no studies have assessed the relationship between biomarkers of cell death in donors and the outcomes after heart transplantation.

The aim of this study was to evaluate the association between inflammatory and cell death biomarkers in DBD heart donors and early post-HTx outcomes, specifically PGD and acute rejection.

## 2. Results

### 2.1. Baseline Characteristics and HTx Process

**Figure A1:**
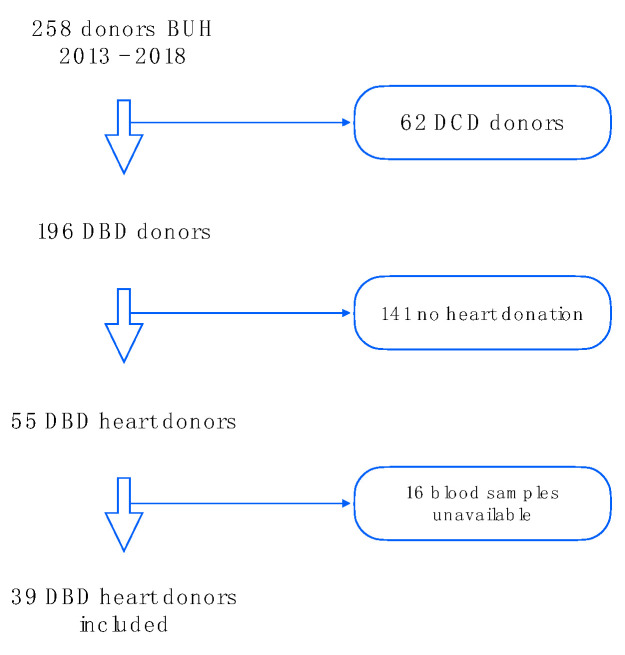
Donor flow chart. BUH: Bellvitge University Hospital; DCD: donation after circulatory death; DBD: donation after brain death.

During the study period, there were 258 cadaveric organ donors. Donations after the circulatory death (DCD) program did not start until 2021 and sixty-two DCD donors were excluded. Of the 196 DBD donors, 55 were heart donors. We excluded 16 patients whose serum samples could not be processed as sample processing was not available on weekends. Finally, 39 heart donors were included in the study (Figure A1). The hearts were distributed according to the national and regional distribution criteria, and all recipients were transplanted in Spain. The organs were transported and preserved using standard cold storage methods (Table S1). 

The main age of DBD donors was 46 years, with 35.9% being women. Most of them were smokers (61.5%) with low prevalence of other classic cardiovascular risk factors. The main cause of death was stroke (33%) followed by subarachnoid hemorrhage (30%) and traumatic brain injury (20%). Related to the ICU complications, more than 50% of patients suffered ventilator-associated pneumonia and 12,8% of patients presented acute kidney injury (Table 1). 

Recipient mean age was 55.3 years, with 17.9% being women. The indication for Htx was ischemic cardiomyopathy in 41% of patients and the prevalence of cardiovascular risk factors was high, with 33% of patients being diabetic. More than 40% of recipients were on chronic inotrope support (15.4%) or MCS (28.2%) at the time of HTx, with more than 35% of patients listed as status 0 priority (Table 2). 

The mean time that elapsed between brain death diagnosis and organ procurement was 14 h. The mean ischemic time was 158 min, and there was widespread use of vasopressors at medium doses (mean maximum noradrenaline dose of 0.31 ± 0.19 mcg/kg/min).

**Figure A2:**
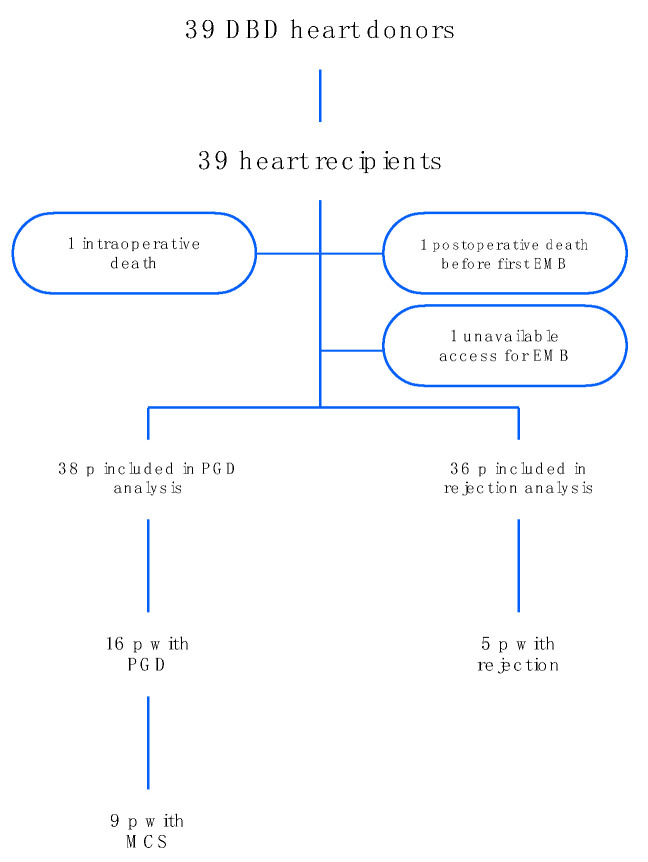
Heart recipients flow chart. EMB: endomyocardial biopsy; MCS: mechanical circulatory support; p: patients; PGD: primary graft disfunction.

Data on PGD were available for 38 patients, because one patient died during surgery due to a surgical complication, which was not secondary to PGD (Figure A2). A total of 16 patients experienced PGD—43.8% had severe LV-PGD, 12.5% had moderate LV-PGD, 18.8% had mild LV-PGD, 6.25% had severe RV-PGD, and 18.6% had either mild or moderate isolated RV-PGD, according to ISHLT 2014 definition [20]. Nine patients required MCS; there were six extracorporeal membrane oxygenation (ECMO) cases, one bi-ventricular assist device (BiVAD) case, one right ventricular assist device (RVAD) case, and one intra-aortic balloon pump (IABP).

Rejection surveillance with endomyocardial biopsy (EMB) during the first month post-HTx was available for thirty-six patients; two patients died before first EMB, and in one patient with congenital heart disease, access to the right ventricle was not feasible. A total of five patients (13.8%) showed a cellular rejection grade of ≥2 according to ISHLT criteria during the first month post-transplant. Antibody-mediated rejection was also explored through immunohistochemistry analysis in the biopsy, yielding negative results for all individuals. All patients received standard immunosuppression with steroids, tacrolimus, and mycophenolate. At least one dose of basiliximab was administered to 33 patients (86.8%). None of the patients received induction treatment with thymoglobulin or plasmapheresis. Mortality during ICU stay was 10% and remained unchanged at 30 days. There was one intraoperative death.

### 2.2. Primary Graft Dysfunction According to Brain Death Donor Profile

No clinical or laboratory parameter from the donor was significantly associated with the development of severe primary graft dysfunction (PGD) in the recipient. Likewise, no correlation was observed between the time from brain death diagnosis to organ procurement and PGD incidence. Thyroid hormone administration in donors also failed to improve outcomes. In contrast, recipients with preoperative mechanical circulatory support (23.5% vs. 32%, *p* = 0.04) or those exposed to longer cardiopulmonary bypass times (115 min [111–140] vs. 137 min [122–205]; *p* = 0.03) exhibited a higher incidence of severe PGD (Table S2).

**Figure 1:**
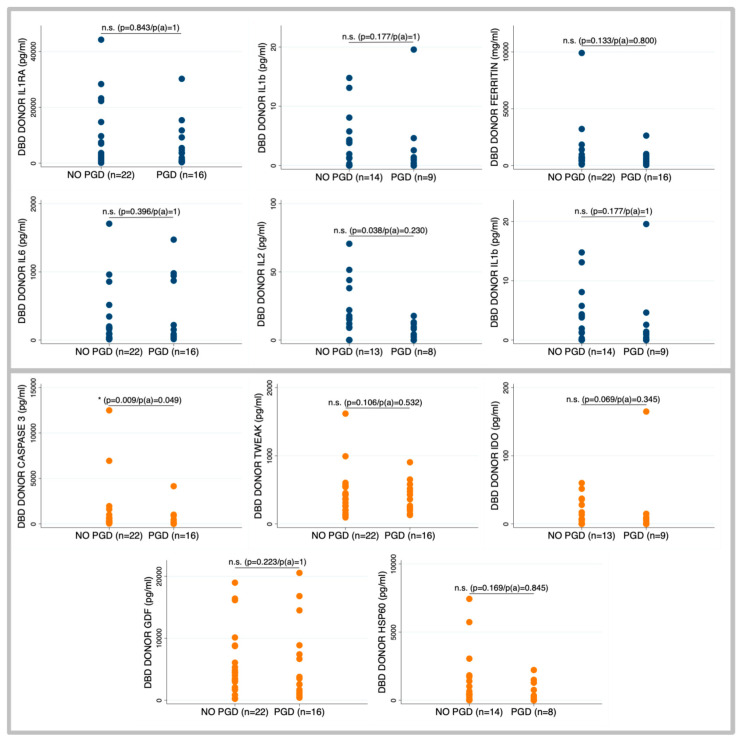
Donor biomarkers on blood samples obtained within the first 24 h after brain death diagnosis. Differences in the levels of biomarkers associated with cell death process and inflammation between patients whose recipients developed primary graft disfunction and those who did not. Comparisons between groups were performed using the Mann–Whitney U test. For multiple comparisons, *p* values were adjusted using the Bonferroni correction, p(a): p adjusted.

Among the biomarkers assessed, Caspase-3 emerged as the strongest discriminator, with significantly lower levels in donors whose recipients developed severe PGD. This association remained statistically significant after adjustment for multiple comparisons, suggesting a potential protective role of apoptotic priming in graft resilience (Figure 1). A similar trend was observed for IL-6 and IL-2, which were also lower in the group of donors whose recipients developed PGD, although these differences were not statistically significant. Similarly, reduced mtDNA/gDNA ratios were noted in donors associated with PGD (10.5 [5.4–24.6] vs. 6.5 [3.3–10.7]; *p* = 0.067) but failed to reach significance (Table 3).

### 2.3. Occurrence of Rejection According to Brain Death Donor Profile

No clinically significant differences in acute cellular rejection were observed in relation to donor age, cause of death, comorbidities, or baseline organ function. Similarly, no differences were found in cardiologic biomarkers or in the use of vasopressors or inotropes (Table S3).

**Figure 2:**
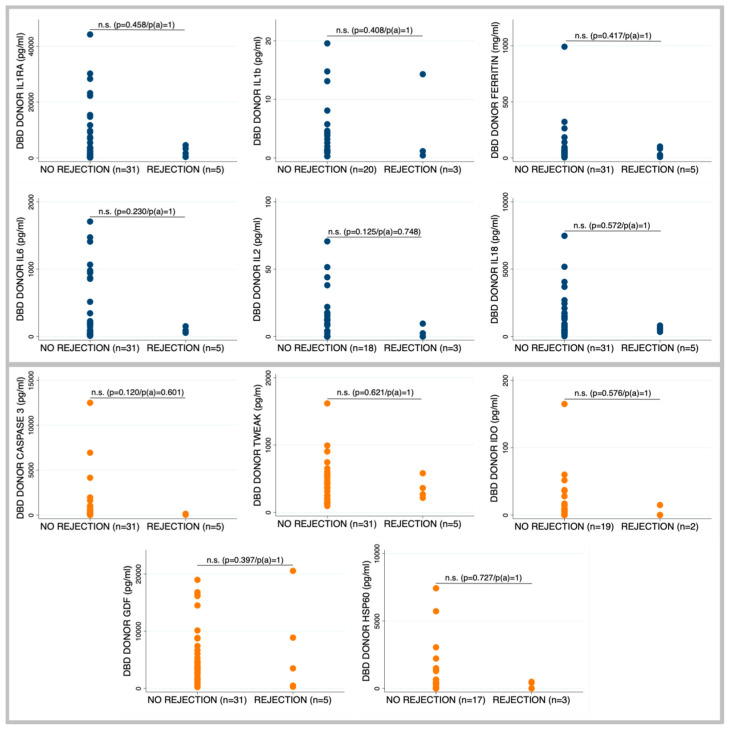
Donor biomarkers on blood samples obtained within the first 24 h after brain death diagnosis. Differences in the levels of biomarkers associated with cell death process and inflammation between patients whose recipients developed acute cellular rejection and recipients who did not develop cellular rejection. Comparisons between groups were performed using the Mann–Whitney U test. For multiple comparisons, *p* values were adjusted using the Bonferroni correction, p(a): p adjusted.

Recipients who developed grade ≥2 cellular rejection received organs from donors exhibited a trend toward lower levels of pro-inflammatory cytokines, particularly IL-2 and IL-6 (Figure 2). Although differences in apoptosis-related biomarkers such as Hsp60 and Caspase-3 did not reach statistical significance, consistently lower levels were noted in donors associated with rejection. A similar non-significant trend was observed in mtDNA/gDNA ratios (7.8 [5.1–25.2] vs. 10.9 [5.9–14.5]; *p* = 0.117), suggesting a potential biological pattern despite a lack of significance (Table 3).

## 3. Discussion

Serum biomarkers have demonstrated considerable promise for clinical use and their application is expanding in some aspects of transplantation such as the non-invasive surveillance of rejection [21]. However, evidence regarding their potential application in donor evaluation and management is still scarce. We present one of the largest studies to date evaluating cell death and inflammation-related biomarkers in DBD heart donors and their association with early clinical outcomes after HTx.

PGD and allograft rejection continue to be major challenges, significantly affecting first-year survival after HTx. In our cohort, severe PGD occurred frequently, with many cases requiring MCS. Despite the incidence of severe PGD, 30-day post-transplant survival remained high (90%), consistent with international registry data [1,2].

To date, predictive models for PGD have shown limited clinical accuracy. In our study, the only donor-related factors associated with PGD were observed in recipients with prior mechanical circulatory support and longer durations of cardiopulmonary bypass, and findings aligned with recent publications [22].

Available evidence also suggests that traditional myocardial injury biomarkers offer limited discriminatory value in donor assessment [23,24] a finding consistent with our results. Similarly, the administration of thyroid hormones in donors showed no improvement in post-transplant outcomes, reinforcing prior observations [25].

Periods of cellular stress drive excessive reactive oxygen species production and mitochondrial dysfunction that causes the mitochondrial permeability transition pore opening and activation of the intrinsic apoptotic cascade [26]. In our cohort, donors whose recipients did not develop severe PGD exhibited a biomarker profile consistent with sublethal mitochondrial stress: they had modestly elevated inflammatory cytokines, higher mtDNA/gDNA ratios, and significantly increased Caspase-3 levels.

Caspase-3 activation can occur via the intrinsic pathway (triggered by mitochondrial outer membrane permeabilization) and the extrinsic pathway (death receptor–mediated) [27]. Higher levels of Caspase-3 and higher mtDNA/gDNA ratios were associated with a lower incidence of PGD. Both elevated mtDNA/gDNA ratio and Caspase-3 suggest controlled mitochondrial permeability and the activation of the intrinsic apoptotic cascade.

In vitro studies showed that sublethal caspase activation has an important role in cardiomyocyte differentiation. Bulatovic et al. demonstrated that caspase signaling promotes the proliferation of cardiac progenitor cells, whereas its inhibition impairs cardiomyogenesis. These findings suggest that controlled apoptotic signaling might play an important role in driving myocardial repair post-injury [28]. Moreover, in vitro evidence also indicates that sublethal mitochondrial signals can trigger inflammation, alerting nearby cells in response to stressors such as chemotherapy or infection [29].

Additionally, recent evidence indicates that caspases, beyond their classical role in apoptosis, may regulate the biogenesis and cargo loading of extracellular vesicles (EVs) even in non-lethal scenarios. These EVs can serve as mediators of cell-to-cell communication, stress adaptation, and immunomodulation [30].

In this context, higher Caspase-3 levels in donors might promote the release of EVs that precondition the graft by modulating the local immune response or enhancing cellular stress resilience. A recent study conducted in pigs demonstrated brain death induced expression of pro-inflammatory and pro-apoptotic markers, leading to the development of right ventricular dysfunction in donors, which could potentially be prevented by tacrolimus [31]. However, there is no clinical study that proves the benefit of using calcineurin inhibitors in the prevention of post-transplant PGD, and previous studies with corticosteroid treatment in donors have yielded unsatisfactory results [32,33].

Taken together, our results suggest that the presence of inflammation, capable of triggering non-lethal caspase activation, could lead to a preconditioning of the myocardial cell before the subsequent stress. This hypothesis may also help explain why the broad inhibition of inflammation might not be the most effective therapeutic strategy in a donor setting.

Limitations: This is a hypothesis-generating study with limited statistical power due to the small sample size. Nevertheless, it represents one of the largest studies to date investigating donor-derived biomarkers in HTx. Although multiple comparisons were performed, appropriate statistical adjustments were applied to mitigate this limitation. Some biomarkers showed missing values inherent to the assay; however, this did not affect Caspase-3, the main focus of the study. Donor management was standardized, as all donors originated from the same institution; however, the multicenter design may have introduced variability in post-transplant care practices, potentially influencing clinical outcomes. Although the histological origin of circulating Caspase-3 was not assessed, previous studies have demonstrated its activation in cardiac tissue from DBD donors [34]. Despite this limitation, the observed association between serum Caspase-3 levels and PGD remains of interest due to its potential clinical applicability.

## 4. Materials and Methods

### 4.1. Patient Selection

We performed a retrospective, multicenter observational study. We included potential heart donors of more than 18 years old, admitted to the Intensive Care Unit of Bellvitge University Hospital between August 2013 and July of 2018. We included all DBD donors with available stored blood samples in order to perform subsequent immunological analysis. The hearts were distributed according to the national and regional Transplant Organization distribution criteria and all centers with corresponding recipients were invited to participate in the study. The study was approved by the Bellvitge University Hospital research ethical committee (number PR066/17; approved on: 11 May 2017).

### 4.2. Data Collection

The epidemiological and clinical characteristics of DBD donors were recorded at the time of brain death diagnosis. Data related to intensive care unit (ICU) admission, including inotrope use and other medications, hemodynamics and biochemical parameters were collected.

The epidemiological and clinical characteristics related to the recipient’s baseline status, as well as data of the postoperative course, which included surgical times, treatments administered during ICU admission and biochemical parameters. PGD was defined and graded according to the 2014 ISHLT consensus statement [35]. The requirement for mechanical circulatory support due to PGD was specifically documented and classified as severe. Recipients with graft failure due to surgical complications, hyperacute rejection, or pulmonary hypertension were excluded from the PGD analysis. Rejection was classified according to the ISHLT definition [36], and we considered clinically significant rejection as the presence of cellular rejection ≥2R or humoral rejection needing bolus steroids ± additional therapies.

### 4.3. Sample Acquisition

The blood samples were obtained from each donor within the first 24 h after brain death diagnosis. Then the samples were stored at least 30 min at room temperature, centrifuged at 1200× *g* for 10 min and then aliquoted into 1.5 mL tubes and stored at −80 °C until immunological analysis.

### 4.4. Multiplex Analysis

The concentrations of interleukins, inflammatory mediators, and cell death markers—including IL-1α, IL-1β, IL-1RA, IL-2, IL-6, IL-8, IL-10, IL-18, IL-33, IFN-δ, TNF-α, TREM-1, CD28, IDO, GDF, MIP-1, MCP-1, C5a, sFas-L, TRAIL, TWEAK, Caspase-3, and Hsp60—were quantified using the ProcartaPlex Human Plex Panel (ThermoFisher^®^, Waltham, MA, USA), following the manufacturer’s instructions. Plates were processed using Luminex MAGPIX^®^ technology (Luminex corporation, Diasorin company, Austin, TX, USA). Results were expressed as median fluorescence intensity (MFI) and converted to pg/mL based on standard curves.

### 4.5. Mitochondrial and Genomic DNA

The free DNA from 100 uL of serum from the heart donors was isolated with the DNeasy blood and tissue extraction kit (Qiagen, Hilden, Germany), following the manufacturer’s instructions. The DNA was eluted in 100 μL of elution buffer, sonicated for 10 min at 38 kHz to fragment the DNA, measured in Qbit4 fluorometer (Thermofisher Scientific) and then the concentration was adjusted to 1–10 ng/mL. The samples were stored at −80 °C until the reverse transcription polymerase chain reaction (RT-PCR) was performed. The RT-PCR for mitochondrial DNA (mtDNA) and genomic DNA (gDNA) was performed according to Ajaz S. et al. [37]. For the mtDNA, we used the forward primer hMitoF3 5′-CACTTTCCACACAGACATCA-3′ and the reverse primer hMitoR3 5′-TGGTTAGGCTGGTGTTAGGG-3′; for the gDNA, we used the forward primer hB2MF1 5′-TGTTCCTGCTGGGTAGCTCT-3′ and the reverse primer hB2MR1 5′-CCTCCATGATGCTGCTTACA-3′. The amplification was performed with the Quantitect Sybr Green PCR (Qiagen 204141) in the CFX95 thermal cycler (BioRad, Hercules, CA, USA) by triplicate, with an initial denaturation at 95 °C for 15 min (1 cycle), followed by denaturation at 95 °C for 10 s, annealing at 60 °C for 30 s, and extension at 72 °C for 1 min and 30 s (40 cycles) and melt curve analysis. Sample concentrations were calculated using a calibration curve based on the linear regression equation y = −mx + b. Measurements are expressed as DNA copy number. The mtDNA/gDNA ratio is presented as a descriptive index.

### 4.6. Data Analysis

Biochemical and clinical donor parameters were associated with recipient outcomes assessed as mortality, rejection, and PGD with or without the requirement of circulatory support. Continuous nonparametric variables were summarized as the median (±IQR), and those that follow a normal distribution as the mean (±SD). Categorical variables were expressed as count and percentage. The groups were compared with the χ^2^ test for categorical variables or Fisher’s exact test in the case of 2 × 2 tables, an analysis of variance (ANOVA) or Student’s *t*-test for continuous variables in data with normal distribution, and the nonparametric Kruskal–Wallis or Mann–Whitney U tests for data that did not follow a normal distribution. All statistical tests and confidence intervals were constructed with a type I error alpha value of 5%. The Bonferroni correction was applied to adjust the significance level in multiple analyses of donors’ inflammatory and cell death biomarkers to minimize the risk of Type I errors. Analyses were performed with Stata software (version 16.1).

## 5. Conclusions

PGD remains a major challenge in HTx. Our findings suggest that non-lethal caspase activation in the donor may precondition myocardial cells, enhancing graft tolerance to subsequent stress. Lower donor serum Caspase-3 levels were linked to severe PGD, indicating a possible protective role of sublethal apoptotic signaling. These results underscore a donor’s critical influence on transplant outcomes and highlight the underexplored potential of donor serum biomarkers for prognostic evaluation and clinical decision-making.

## Figures and Tables

**Table 1:** Brain dead heart donors baseline characteristics, including previous comorbidities and cause of death. Summary of laboratory test results, information related to infections and treatment received during ICU stay.

Donor Baseline Characteristics (N = 39)	
**Comorbidities**	
Age (years)	46.4 ± 12.4
Women, n (%)	14 (35.9)
Diabetes mellitus, n (%)	0
Hypertension, n (%)	6 (15.4)
Dyslipidemia, n (%)	6 (15.4)
Kidney disease, n (%)	0
Lung disease, n (%)	0
Liver disease, n (%)	2 (5.13)
Cardiac arrest, n (%)	5 (12.8)
**Laboratory results**	
Troponin T (ng/L)	156 ± 316
Lactate (mmol/L)	1.66 ± 1.07
Creatinine (µmol/L)	88.2 ± 47.8
Sodium (mmol/L)	148 ± 27.7
PaCO_2_ (mmHg)	38.7 ± 5.98
PaO_2_ (mmHg)	298 ± 122
HCO_3_ (mmol/L)	25.8 ± 3.80
Albumin (g/L)	35.7 ± 7.38
Prealbumin (g/L)	178 ± 101
ALT (U/L)	0.91 ± 1.01
Bilirubin (µmol/L)	12.2 ± 10.3
LDH (U/L)	4.73 ± 1.71
Hemoglobin (g/dL)	11.9 ± 2.67
Leucocytes (10^9^/L)	15.58 ± 6.32
**Cause of death, n (%)**	
Hanging	1 (2.56)
Anoxic encephalopathy	3 (7.69)
Stroke	13 (33.3)
Suicide	1 (2.56)
Subarachnoid hemorrhage	12 (30.8)
Traumatic brain injury	8 (20.5)
**Infections, n (%)**	
Respiratory infection	20 (55.6)
Microbiological isolation	18 (46.15)
**Treatments, n (%)**	
Steroids	6 (15.4)
Levothyroxine	5 (12.8)
Desmopressin	32 (82.1)
Mineralocorticoids	12 (30.8)

Continuous variables are expressed as means ± SD.

**Table 2:** Heart recipients baseline characteristics, including previous comorbidities and pre-HTx situation. Continuous variables are expressed as means ± SD.

Heart Recipient Baseline Characteristics (N = 39)	
Age (years)	55.3 ± 11.2
Height (cm)	164 ± 28.7
Weight (kg)	73.3 ± 15.5
Women, n (%)	7 (17.9)
Diabetes mellitus, n (%)	13 (33.3)
Hypertension, n (%)	12 (30.8)
Dyslipidemia, n (%)	16 (41.0)
Prior smoker, n (%)	19 (48.7)
Chronic kidney disease, n (%)	11 (28.2)
Ischemic cardiomyopathy, n (%)	16 (41.0)
Chronic inotrope support, n (%)	6 (15.4)
Previous MCS, n (%)	11 (28.2)
Emergency 0 status, n (%)	14 (35.9)
Emergency 1 status, n (%)	5 (12.8)
Sensitization, n (%)	0
Time on waiting list (days)	107.2 ± 141.2
INTERMACS profile n (%)	
1	7 (17.9)
2	5 (12.8)
3	6 (15.4)
≥4	21 (53.8)

**Table 3:** Distribution of genomic DNA (gDNA), mitochondrial DNA (mtDNA), and the mtDNA/gDNA ratio in DBD donors according to the occurrence of acute cellular rejection and PGD in recipients. Comparisons between groups were performed using the Mann–Whitney U test and variables are expressed as median [IQR].

Variable	No Rejection	Rejection	* p *	No PGD	PGD	* p *
gDNA	5.8 [1.6–13.7]	11.5 [2.5–27.7]	0.327	5.8 [1.3–13.7]	6.2 [2.5–23.6]	0.534
mtDNA	63.5 [18.4–170.0]	108.6 [28.6–306.1]	0.385	74.4 [18.8–274.4]	26.7 [19.6–143.0]	0.438
Ratio	7.8 [5.1–25.2]	10.9 [5.9–14.5]	0.117	10.5 [5.4–24.6]	6.5 [3.3–10.7]	0.067

## Data Availability

The raw data supporting the conclusions of this article will be made available by the authors on request.

## Supplementary Materials

The following supporting information can be downloaded at: https://www.mdpi.com/article/10.3390/ijms26199434/s1.

## Abbreviations

The following abbreviations are used in this manuscript:

ALT	Alanine aminotransferase
AST	Aspartate aminotransferase
BiVAD	Biventricular assist device
C5A	Complement component 5a
CD28	Cluster of differentiation 28
DCD	Donation after circulatory death
DBD	Donation after brain death
ECMO	Extracorporeal membrane oxygenation
EMB	Endomyocardial biopsy
EV	Extracellular vesicles
GDF	Growth differentiation factor
gDNA	Genomic deoxyribonucleic acid
Hsp60	Heat shock protein 60
HTx	Heart transplant
ICU	Intensive care unit
IFN-γ	Interferon gamma
IL	Interleukin
IL1RA	Interleukin-1 receptor antagonist
INTERMACS	Interagency Registry for Mechanically Assisted Circulatory Support
IP-10	Interferon gamma-induced protein 10
ISHLT	International Society for Heart and Lung Transplantation
IABP	Intra-aortic balloon pump
LDH	Lactate dehydrogenase
MCP-1	Monocyte chemoattractant protein 1
MCS	Mechanical circulatory support
mtDNA	Mitochondrial deoxyribonucleic acid
PGD	Primary graft dysfunction
RVAD	Right ventricular assist device
sFAS-L	Soluble Fas ligand
TNF-α	Tumor necrosis factor alpha
TRAIL	Tumor necrosis factor-related apoptosis-inducing ligand
TREM-1	Triggering receptor expressed on myeloid cells 1
TWEAK	Tumor necrosis factor-like weak inducer of apoptosis
