# Supplementary material for: Caspase-3 in Brain Death Donors Is Associated with Reduced Primary Graft Dysfunction After Heart Transplantation
Source: Int J Mol Sci. 2025 Sep 26;26(19):9434. doi: 10.3390/ijms26199434 (PMC12524385; doi:10.3390/ijms26199434)
Supplement: Supplementary file 1 [file ijms-26-09434-s001.zip › ijms-3720649-supplementary.pdf]

Supplementary Table S1. Final distribution of donor hearts among hospitals in Spain.

Hospital	City	Number of HTx
Hospital Universitario de Bellvitge	Barcelona	20
Hospital de la Santa Creu i Sant Pau	Barcelona	7
Hospital Clinic	Barcelona	3
Hospital La Fe	Valencia	2
Hospital Gregorio Marañon	Madrid	2
Hospital Miguel Servet	Zaragoza	2
Hospital Virgen de la Arrixaca	Murcia	1

Supplementary Table S2. Baseline Characteristics of brain death (DBD) donors according to severe primary graft dysfunction (PGD) in Heart Recipients. Comparisons were performed using Fisher's exact test for categorical variables and Mann–Whitney U test for continuous variables.

DBD donors baseline characteristics according to severe PGD occurrence			
Variable	No severe PGD (N=29)	Severe PGD (N=9)	P
	N(%)/Median[IQR]	N(%)/Median[IQR]	
Age	49.0 [41.0;54.0]	54.0 [46.0;59.0]	0.236
Hypertension	5 (17.2%)	1 (11.1%)	1.000
Diabetes	0	0	
Dyslipidemia	3 (10.3%)	3 (33.3%)	0.131
Smoking	17 (58.6%)	6 (66.7%)	1.000
Cause of death<br>Cardiac arrest<br>Hanging<br>Anoxia<br>Stroke<br>Suicide<br>Subarachnoid<br>hemorrhage<br>[illegible]	 3<br>1<br>1<br>11<br>1<br>8<br>1<br>[illegible]	 1<br>0<br>2<br>2<br>0<br>3<br>0<br>[illegible]	0,635
Troponin (ng/L)	61.0 [14.0;135]	28.5 [18.8;113]	0.761
Creatinine (µmol/L)	58.0 [47.0;83.0]	69.0 [40.0;86.0]	0.837
Sodium (mmol/L)	154 [147;159]	153 [150;156]	0.823
Bilirubin (µmol/L)	9.00 [6.00;13.7]	7.00 [6.00;11.0]	0.558
AST (U/L)	0.55 [0.32;1.29]	0.33 [0.30;0.48]	0.250
Lactate (mmol/L)	1.50 [0.90;2.10]	1.20 [1.00;2.00]	0.877
Hemoglobin (g/dL)	11.4 [9.30;13.6]	12.5 [10.3;12.8]	0.492
Leucocytes (10^9/L)	17.0 (6.6;28.5)	16.7 (9.8;24.2)	0.471
pO2 (mmHg)	273 [194;376]	335 [288;353]	0.655
pCO2 (mmHg)	38.0 [35.0;42.0]	41.0 [37.0;42.0]	0.480
Levothyroxine	3 (17.2%)	0 (0.00%)	0.862

Supplementary Table S3. Baseline Characteristics of brain death (DBD) donors according to clinically significant cellular rejection (≥2R cellular rejection or humoral rejection needing bolus steroids +/- additional therapies) occurrence on heart recipient. Comparisons were performed using Fisher's exact test for categorical variables and Mann–Whitney U test for continuous variables.

DBD baseline characteristics according to cellular rejection occurrence			
Variable	No rejection	Rejection (N=5)	P
	N(%)/Median[IQR]	N(%)/Median[IQR]	
Age	48.0 [41.0;54.5]	49.0 [48.0;58.0]	0.680

Hypertension	4(12.9%)	1(20.0%)	0.549
Diabetes	0	0	
Dyslipidemia	5 (16.1%)	1 (20.0%)	1,000
Smoking	19 (61.3%)	3 (60.0%)	1.000
Cause of death<br>Cardiac arrest<br>Hanging<br>Anoxia<br>Stroke<br>Suicide<br>Subarachnoid hemorrhage<br>Meningitis<br>Traumatic brain injury	 4<br>1<br>2<br>10<br>1<br>9<br>1<br>7	 0<br>0<br>1<br>2<br>0<br>1<br>0<br>1	0.841
Troponin (ng/L)	60.0 [15.0;142]	35.0 [22.0;41.0]	0.340
Creatinine (μmol/L)	71.0 [50.5;98.5]	57.0 [45.0;65.0]	0.337
Sodium (mmol/L)	150 [146;154]	148 [148;154]	0.909
Bilirubin (μmol/L)	9.00 [5.65;13.3]	7.00 [7.00;11.0]	0.909
AST (U/L)	0.48 [0.31;1.12]	0.46 [0.19;0.92]	0.647
Lactate (mmol/L)	1.41 [0.90;1.95]	1.80 [1.10;2.16]	0.583
Hemoglobin (g/dL)	11.4 [9.55;13.2]	12.6 [10.3;14.4]	0.410
Leucocytes (10^9/L)	14.2 (6.6;28.5)	18.0 (16.7;24.2)	0.088
pO2 (mmHg)	273 [192;348]	354 [353;355]	0.037
pCO2 (mmHg)	40.0 [35.0;42.0]	41.0 [38.0;45.0]	0.371
Levothyroxine	5 (16.1%)	0 (0.00%)	0.279
